# Postnatal exposure to ambient air pollutants is associated with the composition of the infant gut microbiota at 6-months of age

**DOI:** 10.1080/19490976.2022.2105096

**Published:** 2022-08-13

**Authors:** Maximilian J. Bailey, Elizabeth A. Holzhausen, Zachariah E. M. Morgan, Noopur Naik, Justin P. Shaffer, Donghai Liang, Howard H. Chang, Jeremy Sarnat, Shan Sun, Paige K. Berger, Kelsey A. Schmidt, Frederick Lurmann, Michael I. Goran, Tanya L. Alderete

**Affiliations:** a Department of Integrative Physiology, University of Colorado Boulder, Boulder, CO, USA; b Department of Pediatrics, University of California San Diego, La Jolla, CA, USA; c Rollins School of Public Health, Emory University, Atlanta, GA, USA; d Department of Bioinformatics and Genomics, University of North Carolina at Charlotte, Charlotte, NC, USA; e Department of Pediatrics, The Saban Research Institute, Children’s Hospital of Los Angeles, University of Southern California, Los Angeles, CA, USA; f Sonoma Technology Inc, Petaluma, CA, USA

**Keywords:** Gut microbiome, infant, development, ambient air pollution, postnatal

## Abstract

Epidemiological studies in adults have shown that exposure to ambient air pollution (AAP) is associated with the composition of the adult gut microbiome, but these relationships have not been examined in infancy. We aimed to determine if 6-month postnatal AAP exposure was associated with the infant gut microbiota at 6 months of age in a cohort of Latino mother-infant dyads from the Southern California Mother’s Milk Study (n = 103). We estimated particulate matter (PM_2.5_ and PM_10_) and nitrogen dioxide (NO_2_) exposure from birth to 6-months based on residential address histories. We characterized the infant gut microbiota using 16S rRNA amplicon sequencing at 6-months of age. At 6-months, the gut microbiota was dominated by the phyla Bacteroidetes, Firmicutes, Proteobacteria, and Actinobacteria. Our results show that, after adjusting for important confounders, postnatal AAP exposure was associated with the composition of the gut microbiota. As an example, PM_10_ exposure was positively associated with *Dialister, Dorea, Acinetobacter*, and *Campylobacter* while PM_2.5_ was positively associated with *Actinomyces*. Further, exposure to PM_10_ and PM_2.5_ was inversely associated with *Alistipes* and NO_2_ exposure was positively associated with *Actinomyces, Enterococcus, Clostridium*, and *Eubacterium*. Several of these taxa have previously been linked with systemic inflammation, including the genera *Dialister* and *Dorea*. This study provides the first evidence of significant associations between exposure to AAP and the composition of the infant gut microbiota, which may have important implications for future infant health and development.

## Introduction

Exposure to air pollutants has been linked with the composition and function of the gut microbiome in adults; however, this relationship has not been examined in infancy.^1–3^ The gut microbiome interfaces with several physiological systems, including but not limited to the immune, endocrine, and the nervous systems.^4–8^ Beyond facilitating several functions across various physiological systems, the composition of the gut microbiome has been associated with several disease states that may have early life origins, including obesity, Crohn’s disease, and type 2 diabetes.^9–11^ Notably, the infant gut microbiome matures within the first 2–3 years of life, which may have long lasting health impacts.^12^ For example, a gut microbiome missing specific bacteria may increase future disease risk through altered immune development, metabolism, and/or development of the enteric nervous system.^13^ Therefore, it is important to examine the early life factors that may impact the development of the gut microbiome, including exposure to inhaled pollutants.

Postnatally, the gut microbiome undergoes rapid and dynamic microbial colonization.^14^ Under normal developmental processes, the newborn gut microbiome includes Enterobacteriaceae and Bifidobacterium.^12^ Throughout the first 6 months of life, the infant gut microbiome becomes largely populated by Firmicutes and Actinobacteria.^12^ Beyond mode of delivery, early life feeding practices, and antibiotic exposure, early life exposure to environmental toxins may also alter the development of the infant gut microbiome.^12,15^ In young adults from Southern California, we have shown that near-roadway and ambient air pollution (AAP) exposure was associated with the composition and functional potential of the gut microbiome.^1,2^ Another study in adults has also shown that the gut microbiota may mediate the associations between AAP and risk for impaired fasting glucose and type 2 diabetes.^3^ These results suggest that exposure to inhaled pollutants may have the potential to impact the human gut microbiome and, consequently, contribute to disease risk. Compared to adults, infants have higher rates of ventilation, which may contribute to higher levels of inhaled exposure to air pollutants.^16^ Early life exposure to air pollution has been shown to negatively impact future health, including respiratory function, cognitive functioning and cardiometabolic health.^17–19^ This may be partly due to the fact that infancy represents a critical developmental window during which many physiological systems, such as metabolism, the visual cortex, the motor cortex, and the immune systems undergo rapid development.^20–23^ Collectively, increased rates of ventilation, rapid physiological growth, and the development of the gut microbiome make early life a critical window where exposure to AAP may have disproportionately deleterious health effects.

Previous work in adults and animal models has shown that exposure to airborne pollutants is associated with the composition and function of the gut microbiome.^1–3,24^ However, there have been no previous studies which have examined the associations between AAP exposure and the composition of the infant gut microbiota to date. Importantly, inhaled pollutants have the potential to reach the gut and may impact the gut microbiome via several mechanisms.^24^ Briefly, inhaled particles can be deposited into the respiratory tract where mucociliary clearance and subsequent ingestion of mucus provides a route for air pollutants to reach the gut. In the gut, particulate matter(PM) and components of traffic emissions, such as carbon derivatives, nitrates, sulfates, and toxic metals, may impact the gut microbial community in the lumen of the gastrointestinal tract.^25^ Additionally, nitrogen dioxide (NO_2_) serves as a marker for near-roadway air pollution, which is a mixture of gases and particles, including black carbon, polycyclic aromatic hydrocarbons, and PM.^26,27^ Solid components of near-roadway air pollution may impact the gut microbiome through deposition in the airways, mucociliary clearance and ingestion, while gaseous components may alter the gut microbiome through endocrine mediated mechanisms.^24,28^ Further, recent studies suggest that AAP may also stimulate the release of corticosteroids and catecholamine production, both of which have been shown to alter the composition of the gut microbiota.^29,30^ Therefore, the aim of the current study was to examine the relationships between postnatal exposure to patriculate matter < 2.5 and 10 microns in aerodynamic diameter (PM_2.5_ and PM_10_)and NO_2_ during the first 6 months of life and the infant gut microbiota at 6 months of age. We hypothesized that exposure to AAP would be associated with the abundance and diversity of gut bacteria.

## Results

We examined 103 Latino infants that were 186 days old on average (range: 164–219 days). Average postnatal exposure to ambient air pollutants (AAP) is shown in Table 1. Overall, we observed a moderate correlation and covariation among these pollutants (**Figure S4** and **Table S5**). For example, log transformed postnatal PM_10_ exposure was moderately correlated with log transformed PM_2.5_ (Pearson r = 0.78; p < .001) and log transformed NO_2_ (Pearson r = 0.52; p < .001). Further, there was moderate correlation between birthweight with log transformed NO_2_ (Pearson r = 0.23; p < .05) and log transformed PM_10_ exposure (Pearson r = 0.20; p < .05). The infant gut microbiota at 6 months of age was first examined by visualizing the top 10 most abundant taxa at the phylum and genus levels (**Figure S3**). At the phylum level, the four most abundant taxa were Bacteroidetes (39%), Firmicutes (24%), Proteobacteria (21%) and Actinobacteria (14%). At the genus level, the four most abundant taxa were *Bacteroides* (29%), an unclassified genus within the family *Enterobacteriaceae* (18%), *Bifidobacterium* (13%) and *Parabacteroides* (7%). We also examined measures gut bacterial alpha-diversity, including Shannon’s index (mean = 2.91, standard deviation = 0.718), richness (mean = 53.6, standard deviation = 26.8), and Faith’s phylogenetic diversity (mean = 7.93, standard deviation = 2.35).

**Table 1.** Characteristics of mother-infant pairs from the Mother’s Milk Study.

Maternal Characteristics	Mean ± SD
Maternal Age (years)	29.7 ± 6.57
Pre-pregnancy BMI (kg/m^2^)	28.1 ± 5.56
Socioeconomic Status (Hollingshead Index)	26.9 ± 11.51
Mode of Delivery	
*Vaginal/Cesarian Section, %Vaginal*	81/22 (79%)
**Infant Characteristics**	**Mean ± SD**
Infant Age (days)	185 ± 8.49
Infant Sex	
*Female/Male, %Female*	56/47 (54%)
Infant Birthweight (kg)	3.38 ± 0.398
Breastfeedings Per Day	3.41 ± 3.36
Life Course Antibiotic Exposure	
*No/Yes, %No*	93/10 (90%)
**AAP Exposure (Birth to 6-Months)**	**Mean ± SD**
PM_10_ (µg/m^3^)	32.8 ± 5.04
PM_2.5_ (µg/m^3^)	13.0 ± 1.89
NO_2_ (ppb)	20.5 ± 4.70

Descriptive characteristics of 103 Latino mother-infant dyads from the Southern California Mother’s Milk Study are reported. Data are reported as means and standard deviations (SD) unless otherwise noted.

### AAP exposure was associated with the gut microbiota

**Figure 1.**
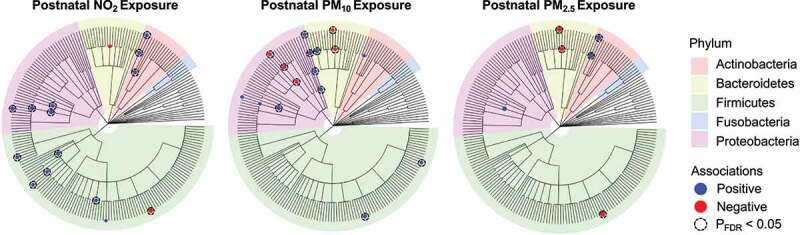
Dendrograms show the associations between NO_2_, PM_10_, and PM_2.5_ exposure with infant gut microbial taxa at 6 months of age using zero-inflated negative binomial regression (ZINBR) analyses. Associations are displayed on a branching tree that shows the phylogenetic relationship between taxa examined in this analysis where branch lengths do not represent evolutionary time. ZINBR models adjusted for infant sex, breastfeeding per day, socioeconomic status, birthweight, and infant age. The direction and magnitude of the association was determined from the incidence risk ratio’s (IRR) distance from an effect estimate which would indicate zero association (IRR = 1). IRRs greater than one represent positive associations (blue), IRRs less than one represent negative associations (red), and the node size denotes the strength of the association. Only associations that were statistically significant at a 10% false discovery rate (P_FDR_ < 0.10) are shown. Nodes framed by a dashed circle indicate statistical significance at a 5% false discovery rate (P_FDR_ <0.05). Each edge in the dendrogram represents various phylogenetic levels (inner to outer circle: kingdom, phylum, class, order, family, genus).

Results from the multivariable zero-inflated negative binomial regression (ZINBR) analysis revealed that postnatal exposure to PM_10_, PM_2.5_, and NO_2_ was associated with the infant gut microbiota at 6 months of age, after adjusting for infant sex, breastfeeding per day, socioeconomic status, birthweight, and infant age (Figure 1, Table 2, and **Table S6**). Here, exposure to PM_10_ and/or PM_2.5_ was positively associated with gut bacterial genera belonging to Actinobacteria, Bacteroidetes, Firmicutes, and Proteobacteria. Specifically, PM_10_ exposure was positively associated with *Dialister* (FDR_BH _= 0.01) and *Dorea* (FDR_BH_ = 0.04) from the phylum Firmicutes, as well as *Acinetobacter* (FDR_BH _= 0.09) and *Campylobacter* (FDR_BH _= 0.03) from the phylum Proteobacteria. Additionally, PM_2.5_ was positively associated with *Actinomyces* (FDR_BH_ = 0.002) from the phylum Actinobacteria. NO_2_ exposure was positively associated gut bacterial genera belonging to Actinobacteria, Firmicutes, and Proteobacteria. This included *Actinomyces* (FDR_BH _= 0.01) from the Actinobacteria phylum, *Clostridium* (FDR_BH _= 0.09), *Enterococcus* (FDR_BH_ = 0.04) and *Eubacterium* (FDR_BH _= 0.04) from the Firmicutes phylum, as well as *Haemophilus* (FDR_BH_ < 0.001) from the Proteobacteria phylum. Further, exposure to PM_10_, PM_2.5_, and/or NO_2_ was inversely associated with gut bacterial genera belonging to Bacteroidetes, Firmicutes, and Proteobacteria. Here, exposure to PM_10_ and PM_2.5_ was inversely associated with the genera *Alistipes* (both FDR_BH_ ≤ 0.02) while exposure to PM_10_ was inversely associated with *Neisseria* (FDR_BH_ < 0.001) from the Proteobacteria phylum. Lastly, exposure to PM_2.5_ and NO_2_ was inversely associated with *Phascolarctobacterium* (FDR_BH_ = 0.005 and FDR_BH _= 0.001, respectively) from the Proteobacteria phylum. These results were largely unchanged in sensitivity analyses that adjusted for mode of delivery (**Table S7**). Next, we found that the associations between AAP and six gut bacterial taxa differed based on infant sex (P_interaction_ < 0.05; **Table S8**). For example, PM_10_ exposure was positively associated with the gut bacterial genus *Acinetobacter* in males (P < .001) but not females (P = .631). Conversely, PM_10_ exposure was positively associated with the gut bacterial genus *Dialister* in females (P = .002) but not males (P = .125). Additionally, we used multivariable linear regression analyses and found that postnatal exposure to PM_10_, PM_2.5_, and NO_2_ was not associated with any of the highly abundant gut microbial taxa (i.e., taxa present in > 95% of samples) as shown in **Table S9**. Finally, there were no significant associations between AAP exposure and measures of alpha- or beta-diversity (i.e., Bray-Curtis Dissimilarity Index and Weighted Normalized UniFrac).

**Table 2.** Infant gut bacterial genera belonging to dominant phyla were associated with postnatal exposure to ambient air pollution (AAP).

Phyla	Actinobacteria	Bacteroidetes	Firmicutes			Proteobacteria		
Genus	*Actinomyces*	*Alistipes*	*Dialister, Dorea*	*Clostridium, Enterococcus, Eubacterium*	*Phascolarcto-bacterium*	*Acinetobacter,Campylobacter*	*Haemophilus*	*Neisseria*
PM_10_ Exposure		-	+			+		-
PM_2.5_ Exposure	*+*	-			-			
NO_2_ Exposure	+			+	-		+	

Summary table displays statistically significant (P_FDR_<0.10) positive (+) and negative (-) associations between postnatal exposure to ambient air pollutants (AAP) and infant gut bacterial genera that were grouped based on the phyla in which they belong. Further, taxa which had a consistent direction of effect were grouped within the same columns. Results shown were based on ZINBR.

### AAP exposure was associated with gut microbial profiles

**Figure 2.**
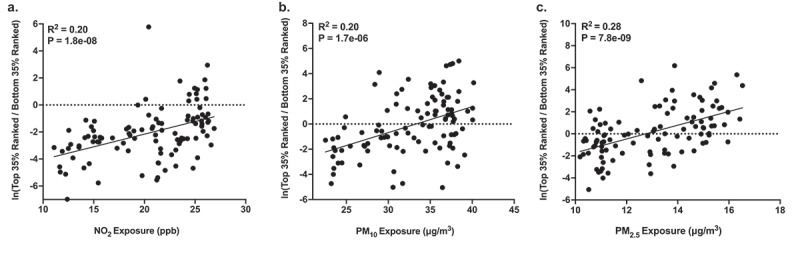
Associations between (a) NO_2_, (b) PM_10_, and (c) PM_2.5_ exposure during the first 6 months of life and differentially ranked log ratios. The differentially ranked log ratios represent the ratio between the top and bottom 35% of sOTUs as ranked based on their association with each pollutant by Songbird (i.e., important taxa).

Using a multinomial regression-based approach, we identified gut bacteria that were associated with postnatal PM_10_, PM_2.5_, and NO_2_ exposure after adjusting for infant sex, breastfeeding per day, socioeconomic status, birthweight, and infant age. As shown in **Table S4**, we examined the top 35% of taxa as ranked by association with exposure to each air pollutant (i.e., normalized to be compositionally robust by taking the log-ratio of the abundances of those taxa with respect to the bottom 35%, and hereafter referred to as ‘*important taxa*’). As shown in Figure 2, we found that AAP exposure was also significantly associated with the normalized abundance of important taxa. For example, NO_2_ exposure was significantly associated with the normalized abundance of important taxa (R^2^ = 0.20, P < .001). Further, PM_2.5_ and PM_10_ exposure were significantly associated with the normalized abundance of important taxa (R^2^ = 0.28, P < .001 and R^2 ^= 0.20, P < .001, respectively). For each exposure, we then examined the classification of these sub-operational taxonomic units (sOTUs) at the genus level and noted specific taxa that were contained within the groups of important taxa for PM_10_ (n = 4), PM_2.5_ (n = 3), and NO_2_ (n = 6), which were also identified in the ZINBR analyses. These genera belonged to the phyla Actinobacteria, Firmicutes and Bacteroidetes. Of the 13 genera identified by both ZINBR and multinomial analyses, all but one displayed the same direction of association with AAP (**Table S10**).

## Discussion

Our results show that increased exposure to AAP during the first 6 months of life was associated with the composition of the infant gut microbiota across multiple taxonomic levels at 6 months of age after adjusting for potential confounders such as infant sex, breastfeedings per day, socioeconomic status, birthweight, and infant age. Importantly, this analysis represents the first study to examine these associations in infancy. Further, this study found several differential associations between AAP exposure and the gut microbiota based on infant sex. These results, along with other epidemiological and animal studies, suggest that environmental exposures such as AAP may impact the human gut microbiota.^24^ These findings have significant public health relevance since 99% of the world’s population is estimated to live in areas where air quality guidelines are not met.^31^

The gut microbiome develops during the first 2–3 years of life.^32^ During this time, the gut microbiome shifts from being dominated largely by *Bifidobacterium* (Actinobacteria) to Bacteroidetes and Firmicutes.^33^ Previous studies in adults have shown that exposure to air pollutants is associated with the composition and functional potential of the gut microbiome.^1–3^ In the current study, we found that measures of AAP were negatively associated with the microbial family *Rikenellaceae*, whereas a previous study in young adults found that near-roadway air pollution exposure was positively associated with the abundance of *Rikenellaceae*.^2^ Additionally, in previous human studies, exposure to particulate matter and NO_2_ was inversely associated with measures of alpha-diversity in some but not all studies.^1–3^ Whereas, in the current study, AAP exposure was not associated with measures of alpha-diversity or beta-diversity. These differential findings may be due to compositional dissimilarities in gut bacterial taxa in early life compared to adults, where the mature gut is more diverse and largely populated by Firmicutes and Bacteroidetes. Additionally, regional differences in the composition of ambient and near-roadway air pollution may contribute to differential effects on microbial communities irrespective of life stage.^34–36^ Despite this, several of the associations that we observed among infants overlap with findings from a previous study among adults. For example, we found that exposure to PM_2.5_ was associated with *Phascolarctobacterium* and *Dorea*. Among adults, species within these genera have been shown to mediate the associations between PM_2.5_ exposure and type 2 diabetes.^3^ In the current study, we also found that the associations between AAP exposure and six gut bacterial taxa differed by infant sex. This is consistent with previous work that found that the associations between arsenic exposure and the gut microbiome differed among male and female infants.^37^ These differential associations may be due to sex based differences in the structure of the gut microbiome and/or gastrointestinal physiology.^38–40^

The current study examined the associations between multiple ambient air pollutants and the infant gut microbiota, including PM_2.5_, PM_10_, and NO_2_. During infancy, age, breastfeeding, introduction of solid food, and mode of delivery have each been shown to impact the development of the gut microbiome.^33,41–43^ Results from our study suggest that AAP exposure may also be an important factor in the development of the gut microbiome. Within Bacteroidetes, higher postnatal exposure to PM_2.5_ was associated with a lower abundance of the family *Rikenellaceae* and the genus *Alistipes*. This suggests that PM_2.5_ exposure may impair the normal transition from *Bifidobacterium* (Actinobacteria) to Bacteroidetes as one of the dominant taxa. Exposure to PM_10_ was positively associated with bacteria belonging to the phylum Firmicutes, including the genera *Dorea* and *Dialister*. Similarly, multiple gut bacteria belonging to the Firmicutes phylum were positively associated with exposure to NO_2_ at the order, family, and genus levels, which included the genera *Clostridium, Enterococcus, Phascolarctoba-cterium* and *Eubacterium*. Additionally, NO_2_ exposure was positively associated gut bacteria belonging to Actinobacteria at the order, family and genus levels that included the genus *Actinomyces*. These findings suggest that NO_2_ and PM_10_ exposure may shape the developing gut microbiota via the early establishment of Firmicutes and Actinobacteria as dominant gut bacterial phyla. Such impacts on the developing gut microbiome may have important implications for infant health and development. For example, a higher ratio of Firmicutes to Bacteroidetes has been associated with obesity in adults and animal studies.^44^

At the genus level, we found that AAP exposure was associated with bacteria that have been linked to adverse health outcomes as well as those known to produce short-chain fatty acids (SCFAs). For example, PM_10_ exposure was positively associated with the abundance of *Campylobacter*, which has been linked with gastroenteritis.^45^ Further, PM_2.5_ and NO_2_ exposure was negatively associated with the abundance of the genus *Phascolarctobacterium*, which has been found to produce high levels of SCFAs.^46^ SCFAs are a major microbial metabolite that has been linked with gut barrier integrity, metabolic and cardiovascular health, as well as gut-brain communication and the maintenance of the blood brain barrier.^47–49^ Alterations to SCFA producing microbes have the potential to impact SCFA availability in the body and play a role in the pathophysiology of various disease states (e.g., inflammatory bowel diseases, obesity, cancer, Parkinson’s disease and celiac disease).^50–53^ Further, the genera *Dialister, Dorea* and *Alistipes* have previously been linked to negative health outcomes such as systemic inflammation, cancer, multiple sclerosis and mental health in adults.^54–57^ We found PM_10_ to be positively associated with *Dialister* and *Dorea*, while PM_10_ and PM_2.5_ were inversely associated with *Alistipes*. However, it is difficult to draw conclusions given that the health effects of these taxa have not been examined in infancy. Lastly, we observed that postnatal PM_2.5_ and PM_10_ exposure had overlapping associations with gut microbial taxa. For example, five of the seven taxa which were associated with PM_2.5_ were similarly associated with PM_10_, potentially because PM_2.5_ makes up a subfraction of PM_10_. Conversely, NO_2_ exposure was associated with 10 taxa that were not associated with PM_2.5_ or PM_10_. This may be due to the fact that NO_2_ is a marker for near-roadway air pollution, which has a different chemical composition than ambient particulate matter.^58^ These differential chemical constituents likely have distinct toxicological mechanisms by which they interact with the gut microbiome. For example, gaseous pollutants have been shown to disrupt endocrine function, whereas components of particulate matter (e.g., heavy metals) may exert direct microbiocidal effects.^25,28,30^ When considered in conjunction with previous work, the current findings seem to suggest that the AAP induced alterations to the gut microbiome may alter human health through inflammatory pathways and altered microbial metabolite production.

Whereas this study provides the first evidence of important associations between postnatal AAP exposure and the developing infant gut microbiota at 6 months of age, there are several study limitations that should be considered. First, we were unable to examine longitudinal associations between AAP and the developing gut microbiome. However, at the completion of infant follow-up, we plan to investigate the associations between AAP with the developing gut microbiome in this cohort. This study also examined associations between AAP and the gut microbiota in a low-income Latino population from Southern California, which may limit generalizability to other populations. However, Latino communities in the US experience a disproportionate level of AAP exposure that may contribute to health disparities observed in an understudied and at-risk population.^59^ Further, we characterized the infant gut microbiota using 16S rRNA amplicon sequencing, which does not allow for reliable taxonomic characterization of gut bacterial taxa below the genus level.^60,61,62,63^ Within all microbial sequencing studies, there is the potential for contamination or index hopping. For this reason, we noted gut bacterial taxa that were associated with AAP exposure and present in our negative controls. However, these taxa have not been identified as common kit contaminants.^64^ Infant AAP exposure was estimated using residence based spatial interpolation, which may introduce error to the classification of exposure estimates when compared to personal monitoring. Yet, such exposure misclassification is likely random and would thus bias our results to the null.^65^ Additionally, without PM speciation data, we were unable to determine if specific components of AAP were more strongly associated with the infant gut microbiota. Future studies should examine the composition of AAP to further elucidate what mechanisms may functionally and toxicologically underlie these associations. Beyond this, future work should incorporate animal and in vitro studies to examine the specific mechanisms by which AAP alters microbial communities. Additionally, tools such as fecal microbiota transplantation can be used to examine the potential physiological implications of AAP-inducted alterations to the gut microbiome.

There were also several statistical limitations of the current study. While all statistical analyses were adjusted for multiple hypothesis testing using a 10% FDR, we cannot rule out the possibility of false positives. Therefore, results from this study should also be viewed as hypothesis generating for future longitudinal investigations. Due to normal developmental processes, we observed a high prevalence of low abundant taxa in the infant gut. For this reason, we used three complementary analytical approaches to examine the associations between postnatal AAP exposure and the infant gut microbiota at 6 months of age. These methods included multivariable linear models, ZINBR analysis, and multinomial regression models that adjusted for important early life factors that were identified based on our directed acyclic graph (DAG), including infant sex, breastfeeding per day, socioeconomic status, birthweight, and infant age. Overall, taxa found to be associated with AAP in the ZINBR analyses were also identified in the differential abundance (i.e., Songbird multinomial regression) analysis and largely displayed the same direction of association. While ZINBR analyses are sensitive to false positives and model diagnostics can be subjective, the concordance in findings between the differential abundance analyses and ZINBR analyses help to alleviate these concerns. Based on our DAG, mode of delivery and early life antibiotic treatment were not identified as potential confounders in the relationship between air pollution exposure and the infant gut microbiota. Nevertheless, due to the importance of these factors in the context of the gut microbiota, we sought to perform additional sensitivity analyses that included adjustment for these variables. Overall, we found that our results were largely unchanged after adjusting for mode of delivery. However, in the current study we were unable to adjust for antibiotic exposure since 90% of infants did not receive antibiotics in the first year of life.

In conclusion, our results demonstrate that increased exposure to PM_2.5_, PM_10_, and NO_2_ in the first 6 months of life was associated with the abundance of several infant gut bacterial taxa belonging to the Bacteroidetes, Firmicutes, Proteobacteria, and Actinobacteria phyla. While previous work has established associations between air pollution exposure and the gut microbiome in adults, this study represents the first characterization of the relationships between postnatal exposures to air pollutants and the infant gut microbiota. Many of the infant gut bacterial taxa that were found to be associated with postnatal AAP exposure have previously been linked with adverse health outcomes such as systemic inflammation, gastroenteritis, multiple sclerosis, and mental health disorders. Further, several of the identified taxa are involved in the production of important gut microbial derived metabolites (e.g., SCFAs) that play an integral role in human physiology. These results, along with other epidemiological and animal studies, suggest that exposure to air pollutants may impact the gut microbiome in early life, which may have implications for human development, health, and physiology.

## Patients, materials and methods

### Study design

Participants were recruited from the Southern California Mother’s Milk Study, which is examining the associations between breast milk factors and infant growth and the gut microbiota in Latino mother-infant pairs.^66^ At the time of analysis, 103 of the 219 mother-infant pairs had complete postnatal AAP exposure data as well as assessment of the gut microbiota at 6-months of age (**Figure S1**). These 103 infants were recruited between 2016–2017 from clinics in Southern California. On average, those included in the current analysis did not significantly differ on any important baseline characteristics except for average AAP. Specifically, AAP exposure was higher among participants that were included in the current analysis than those who were excluded (**Table S1**). Inclusion criteria for the Mother’s Milk Study included healthy term singleton births, first time mothers over the age of 18 years, mothers and fathers with self-reported Hispanic/Latino ethnicity, and an intent to breastfeed for at least 3 months postpartum. Exclusion criteria included medical diagnosis that may affect metabolism, nutritional status, and mental or physical health. Additionally, participants were excluded if they were taking medication which may affect body weight/composition, insulin resistance, or lipid profiles. Further, current smoking or recreational drug use, pre-term births, low birthweight, and fetal abnormalities were exclusion criteria. Written informed consent was obtained from all participants and the study protocol was approved by the University of Southern California, Children’s Hospital Los Angeles, and the University of Colorado Boulder Institutional Review Boards.

### Study visits

As part of the ongoing Mother’s Milk Study, mother-infant pairs are brought in for clinical assessments at 1, 6, 12, 18, and 24-months of age as previously reported.^67^ The Mother’s Milk Study is currently ongoing and not all participants have completed all clinical assessments. Maternal weight was measured to the nearest 0.1 kg (Tanita BC-549 Plus Ironman Body Composition Monitor) and standing height was measured to the nearest 1 mm (Seca 126, Seca GmBH & Co. KG) to calculate body mass index (BMI). Maternal pre-pregnancy BMI was based on self-reported height and weight prior to pregnancy. Infant weight was measured in duplicate to the nearest 5 g by net difference of the mother with and without baby on a Tanita scale. Birth weight (kg) and length (cm) were obtained from hospital records. Infant length was measured to the nearest 1 mm using an infantometer. Infant breastfeedings per day were based on questionnaire data with the following answer choices: 0–1, 1, 2, 3, 4, 5, 6, 7, and ≥8 breastfeedings per day. Briefly, 0–1 breastfeedings per day were assigned to 0 breastfeedings per day, 2–7 breastfeedings per day were assigned to their reported values and ≥8 breastfeedings per day were assigned a value of 8. Lastly, information regarding parental occupation and education was used to examine individual socioeconomic status based on a modified version of the four factor Hollingshead Index as previously reported.^67,68^

### Ambient air pollution exposures

Time weighted average AAP exposure was examined during the first 6 months of life. Exposures included PM_2.5_, PM_10_ as well as NO_2_ which was used as a marker for near-roadway air pollution. Detailed residential address histories (including birth to 6-months of age) were determined at the first study visit and geocoded at the street level using the Texas A&M Geocoding Services (http://geoservices.tamu.edu/Services/Geocode/). Monthly averages of ambient pollutant exposures were estimated from the U.S. Environmental Protection Agency’s Air Quality System (AQS, http://www.epa.gov/ttn/airs/airsaqs), which records hourly air quality data from ambient monitoring stations. Spatial interpolation of up to four of the closest monitoring stations within 50 km of infant homes was performed via an inverse distance-squared weighting (IDW2) algorithm. This method has been demonstrated to be robust to leave-one-out validation for the same data source in California with R^2^ values of 0.73, 0.53, and 0.46 for NO_2_, PM_2.5_, and PM_10_, respectively.^69^ Postnatal exposure was modeled based upon the cumulative average exposure from birth to the 6-month visit (mean exposure day: 185.3, range: 164–219 days). PM_2.5_ and PM_10_ are reported in micrograms per cubic meter (μg/m^3^) and NO_2_ is reported as parts per billion (ppb).

### Gut microbiota

As previously reported, infant stool samples were collected at 6-months postpartum using OMNIgene GUT kits (DNA Genotek, Ottawa, ON, CAN) and a subset have undergone 16S rRNA sequencing.^70^ Briefly, DNA was extracted and the bacteria/archaeal 16S rRNA gene sequenced using the 515/806 barcoded primer pair (515 F [Parada]): GTGYCAGCMGCCGCGGTAA, 806 R [Apprill]: GGACTACNVGGGTWTCTAAT), standardized in accordance with the Earth Microbiome Project.^71^ A ~150-bp fragment was sequenced including variable region 4 (V4) of the 16S rRNA gene. All samples were amplified in triplicate and then pooled into a single sample. Amplicons from each sample were then run on agarose gel to verify the presence of PCR product.^71^ The 515/806 barcoded primer pair has previously been used for cross-cultural analysis, which included infants.^12^ Paired-end (2x150bp) next-generation sequencing was performed using the Illumina MiSeq platform available at the Institute for Genomic Medicine at the University of California (UC) San Diego.^72^ Demultiplexed files were processed using Qiita (https://qiita.ucsd.edu).^73^ Sequences were trimmed to a length of 150-bp, and Deblur was used to remove suspected error sequences and assign amplicon sequence variants called sOTUs.^74^ Subsequently, a feature-table was generated with counts of each sOTU for each sample. To generate a phylogeny, Deblur tag sequences were inserted into the GreenGenes 13_8 backbone phylogeny using SATÉ-enabled phylogenetic placement (SEPP), and all sOTUs not placed were removed from the feature-table.^75^ Negative controls (blanks) and extraction controls were included throughout the amplification and sequencing of samples. The average read depth of the blanks was 1,336 reads as compared to an average read depth of 19,822 for infant stool samples. Taxa present in the blanks were not excluded from the analysis as we were unable to determine if taxa originated from contamination or index hopping.^62^ Given this, we calculated a ratio between the average counts of individual taxa across the blanks to the average counts across the infant samples. The taxa reported in these analyses that exceeded a 1:10 ratio were the order Bacillales, the families *Rikenellaceae* and *Paraprevotellaceae*, and the genera *Dialister* and *Alistipes.*

### Statistical analyses

#### Analytical approach

Descriptive statistics, including means and frequencies of key variables were examined. Overall, three separate and complementary analytical approaches were used to examine the associations between AAP and the infant gut microbiota. ZINBR was the primary analysis performed, as it accounts for over dispersion and a high proportion of zeros present within these data. Complementary analyses were performed to validate the results of the ZINBR as well as to examine aspects of the gut microbiota that cannot be captured via a ZINBR. For example, Songbird analyses account for the compositional nature of microbiome data and use a multinomial regression model to estimate differential rankings for features based on their abundances with respect to model variables. Thus, Songbird analyses were used to provide insight into microbial profiles that may be associated with AAP exposure and to also validate the findings from ZINBR. In addition to these analytical approaches, we also utilized multivariable linear regression to examine highly abundant taxa and alpha-diversity metrics, since these data structures are not appropriate for ZINBR. Finally, Mantel tests were used to evaluate if AAP was associated with measures of beta-diversity (i.e., similarity and dissimilarity matrices). All models were adjusted for infant sex, breastfeedings per day, socioeconomic status, birthweight, and infant age, which was based on a DAG (Figure S2). All participants had complete data for the aforementioned covariates. Race/ethnicity was not controlled for within our statistical models as all participants were Latino. Models which displayed a significant association between AAP exposure and individual taxa were further examined to determine if these associations differed by infant sex by including an interaction term (air pollutant * infant sex). In the case that this interaction term was significant, a stratified analysis was conducted. P-values from all statistical analyses were adjusted for multiple hypothesis testing using a false discovery rate (FDR) of 10% with the Benjamini-Hochberg (BH) procedure. This threshold for statistical significance was determined to increase our statistical power, while also acknowledging that this procedure may be an overadjustment since gut microbial abundances are correlated and the BH procedure assumes independence of tests.^76^ Lastly, the current analysis will be used to generate hypotheses for future investigations related to air pollution exposure and the developing infant gut microbiome in all study participants once follow-up of the Mother’s Milk Study is completed. All statistical analyses were conducted using QIIME2 v.2020.11 and R (Version 4.1.1). Some figures were produced using Prism (GraphPad Version 9.2.0).^77^

#### Gut bacterial diversity

To normalize for sequencing depth, samples were rarefied to a standard read depth of 10,000, which resulted in two samples being dropped. We quantified alpha-diversity (i.e., Shannon’s index, richness, and Faith’s phylogenetic diversity) and beta-diversity (i.e., Bray-Curtis Dissimilarity Index and Weighted Normalized UniFrac) via QIIME2.^77^ Associations between AAP and estimates of alpha- and beta-diversity were interpreted via linear regression models and a Mantel test, respectively. For alpha-diversity metrics, linear regression models adjusted for infant sex, breastfeedings per day, socioeconomic status, birthweight, and infant age.

#### Linear regression

Multivariable linear regression analysis was used to examine the associations between postnatal ambient air pollution exposure and the relative abundance of 25 highly abundant gut microbial taxa (**Table S2**), which were present in more than 95% of the raw sample counts. Relative abundance was log transformed (**Equation 1**) to satisfy the assumptions of linear regression.^78^ Specifically, relative abundance was log transformed to better meet the assumption of normality and homoscedasticity, which were examined via Q-Q plots and by plotting fitted values against standardized residuals, respectively. All models adjusted for the variables identified as potential confounders by a DAG (Figure S2).

**Equation 1**. General Equation for Log Transformed Relative Abundance

$$Log_{10}\left(\left[\frac{Raw\;count\;in\;sample\left(i\right)}{\#\;of\;sequences\;in\;sample\left(i\right)}*Average\;number\;of\;sequences\;per\;sample\right]+1\right)$$

#### Zero-inflated negative binomial regression

Due to normal developmental processes, there were several taxa that were not present in a high proportion of samples. Therefore, ZINBR analysis (PSCL R package) was used to examine the associations between AAP and the abundance of gut microbial taxa after removing rare taxa (i.e., those present in <10% of samples).^79,80^ Briefly, this technique models the abundance of microbes as a mixture of two components: a negative binomial count distribution and a point-mass at zero. For ZINBR models, raw counts were used as the outcome of interest and models were offset by the total sample sequence reads to account for differences in sequencing depth between samples. A zero-inflated model was used to account for overdispersion and two distinct zero-generating processes, one of which is technical and potentially due to sampling error, the other is biological. Additionally, ZINBR was selected due to the potential occurrence of excess zeros due to low abundance taxa in early life as well as potential sampling error. Specifically, ZINBR was chosen over other generalized linear models (e.g., negative binomial regressions and zero-inflated Poisson regression) due to the presence of a high proportion of zeros between 49% and 73% at each taxonomic level (**Table S3**), and overdispersion of microbial counts. In the current analysis, there were an average of 55 microbial taxa with zero counts. Additionally, the average predicted probability of an observation being an excess zero was 0.34. This high proportion of zeros, coupled with significant Vuong tests, suggest that these zero inflated count regression models are an improvement over standard negative binomial models. Specifically, Vuong tests, performed as a sensitivity analysis, revealed significant raw, Akaike information criterion (AIC) and Bayesian information criterion (BIC) corrected and positive z-statistics when individual ZINBRs were compared to standard negative binomial regressions. This suggests that these zero-inflated models have better model fit as compared to non-zero-inflated models.^81,82^ Within our analysis, this technique models the abundance of microbes in the count portion of the model as well as the presence of excess zeros. Incidence risk ratios (IRRs), which represent the estimated cumulative incidence for a one-unit increase in each AAP exposure, were estimated for microbes based only on the negative binomial distribution. The average number of zero count taxa and the average predicted probability of an observation being an excess zero at each taxonomic level can be found in **Table S3**. The “estimated excess zero” sample points were not included in the negative binomial portion of the model. Several additional steps were taken for sensitivity analysis. Predicted values were compared to standardized residuals and theoretical normal quantiles were compared to sample quantiles (Q-Q plots). Further, outliers, with respect to microbial counts, were filtered from the data-set and truncated models were compared to full models to determine if significance was retained. Overall, three statistically significant associations between gut bacteria and AAP were not reported due to being driven by outliers with respect to microbial counts or having Q-Q which varied significantly from the expected distribution. The negative binomial portion of the model adjusted for infant sex, breastfeedings per day, socioeconomic status, birthweight, and infant age as identified by our DAG. The zero inflated portion of the model adjusted for AAP exposure, age, birthweight, and breastfeeding since these variables were thought to potentially contribute to the occurrence of a non-natural zero (e.g., sampling error). Although mode of delivery was not identified as a traditional confounder via our DAG, sensitivity analysis was performed where models additionally adjusted for mode of delivery. This additional sensitivity analysis was performed due to existing literature that suggests that mode of delivery is significantly associated with the composition of the infant gut microbiome. Dendrograms were created using the ggtree R package to summarize the associations between AAP exposure and the abundance of gut bacterial taxa based on ZINBR models (Figure 1).^83^

#### Differential abundance testing

Songbird (v1.0.3) and Qurro (0.7.1) were used to calculate and examine the differential ranks of sOTUs associated with AAP exposure.^84,85^ Briefly, Songbird accounts for the compositional nature of microbiome data and uses a multinomial regression model to estimate differential rankings for features based on their abundances with respect to model variables. The full list of feature rankings from this analysis are included in **Table S4**. Using Qurro, we then selected the top- and bottom 35% of ranked sOTUs, which corresponds to those 35% of taxa that are most- or least-associated with each AAP exposure. Based on this selection, all 103 samples were included when examining NO_2_ and PM_10_, while one sample was excluded when examining PM_2.5_ exposure. For visualization purposes and to perform hypothesis testing, we then examined the log-ratio (**Equation 2**) of the same microbes with each air pollutant using univariate models and R-squared (R^2^) values.

**Equation 2**. General Equation for Differentially Ranked Log Ratio

$$ln\left(\frac{Top\;35\%\;Differentially\;Ranked\;Taxa}{Bottom\;35\%\;Differentially\;Ranked\;Taxa}\right)$$

## Supplementary Material

Supplemental Material

## Data Availability

The data that support the findings of this study are available on request from the corresponding author, TLA. The data are not publicly available, as they contain information that could compromise the privacy of participants included in this study.
