# Supplementary material for: Postnatal exposure to ambient air pollutants is associated with the composition of the infant gut microbiota at 6-months of age
Source: Gut Microbes. 2022 Aug 13;14(1):2105096. doi: 10.1080/19490976.2022.2105096 (PMC9466616; doi:10.1080/19490976.2022.2105096)
Supplement: Supplemental Material [file KGMI_A_2105096_SM5394.zip › SFigures_Tables_06June22.docx]

**Supplemental Figures:**

**Supplemental Figure 1.** Participants from the Southern California Mother’s Milk Study that were Included in the Current Analysis

**Supplemental Figure 1.** At the time of the current analysis, 220 mother-infant pairs were enrolled in the Mother’s Milk Study. Those excluded from the current analysis included those mother-infant pairs that had not yet completed the 6-month visit (n= 17), samples that had not yet undergone the 16S rRNA amplicon sequencing for assessment of the gut microbiota (n= 98), and one for moving outside of California following the 1-month visit (n = 1). Lastly, participants were also excluded due to missing information regarding socioeconomic status (n = 1).

**Supplemental Figure 2.** Directed Acyclic Graph


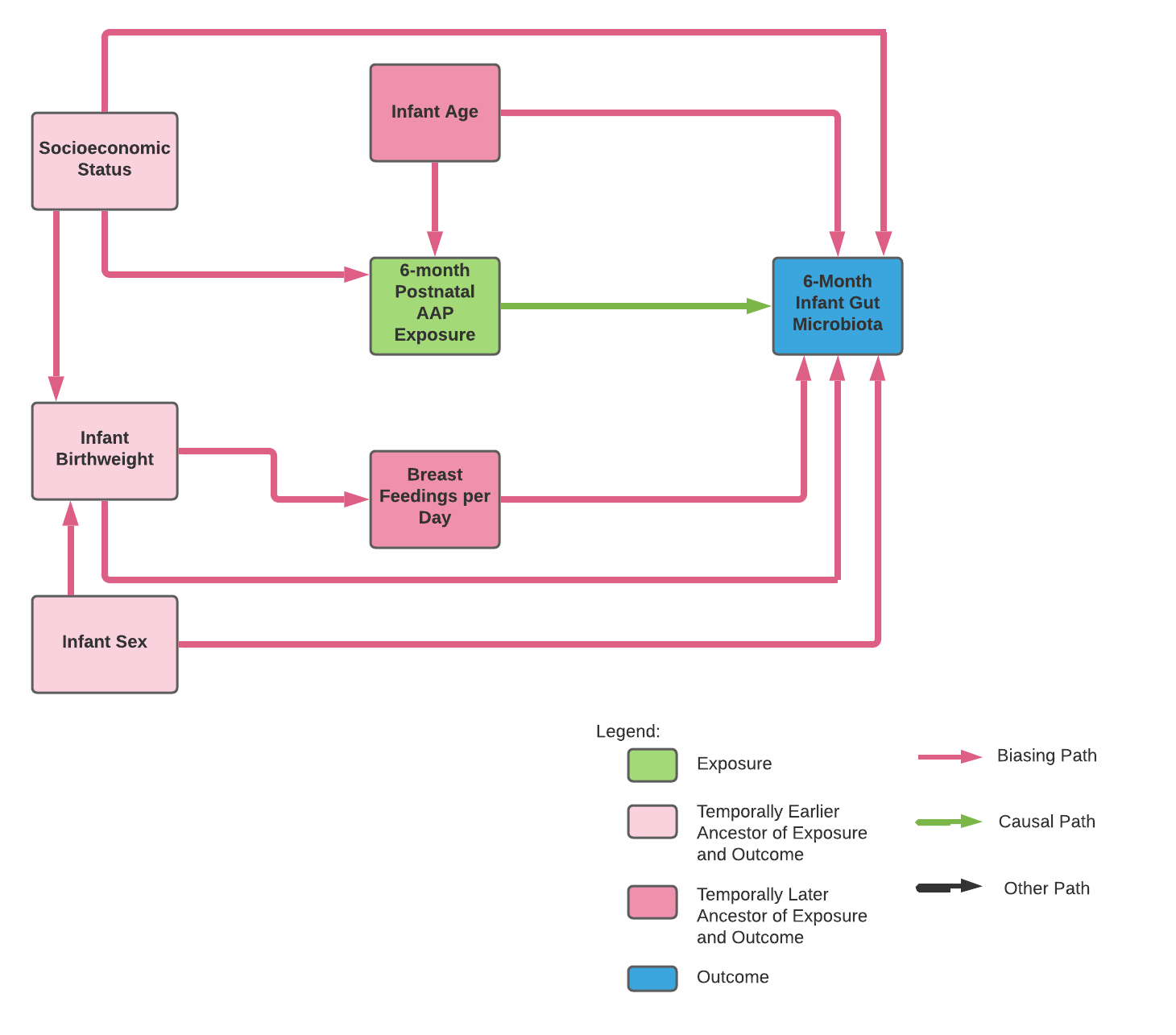


**Supplemental Figure 2.** A directed acyclic graph (DAG) was developed based on casual relationships determined from review of relevant literature. The green node represents the exposure, and the blue node represents the outcomes. The light pink nodes represent adjusted covariates, which temporally precede the exposure. The dark pink nodes represent adjusted covariates that occur temporally after the exposure. Lastly, the pink arrows represent biasing pathways and green arrows represent causal pathways.

**Supplemental Figure 3.** Composition of the Infant Gut Microbiota at 6-Months of Age

**Supplemental Figure 3.** Bar graphs display the relative abundance of the 10 most abundant taxa at the phylum and genus level. Taxa that were not among the 10 most abundant are reported as “Other”.

**Supplemental Tables:**

**Supplemental Table 1.** Characteristics of Mother-Infant Pairs in the Current Analysis and the Full Mother’s Milk Study Cohort

|  | **Analytical Dataset**  **n = 103** |  | **Excluded Dataset**  **n = 100** **^†^** |  |
| --- | --- | --- | --- | --- |
| **Maternal Characteristics** | **Mean ± SD** |  | **Mean ± SD** |  |
| Maternal Age (years) | 29.7 ± 6.57 |  | 29.0 ± 5.91 |  |
| Pre-pregnancy BMI (kg/m^2^) | 28.1 ± 5.56 |  | 29.3 ± 6.23 |  |
| Socioeconomic Status (Hollingshead Index) | 26.9 ± 11.51 |  | 26.3 ± 12.9 |  |
| Mode of Delivery |  |  |  |  |
| *Vaginal/Cesarian Section, %Vaginal* | 81/22 (79%) |  | 70/27 (70%) |  |
| **Infant Characteristics** | **Mean ± SD** |  | **Mean ± SD** |  |
| Infant Age (days) | 185 ± 8.49 |  | 186 ± 9.66 |  |
| Infant Sex |  |  |  |  |
| *Female/Male, %Female* | 56/47 (54%) |  | 52/48 (52%) |  |
| Infant Birthweight (kg) | 3.38 ± 0.40 |  | 3.40 ± 0.43 |  |
| Breast Feedings Per Day | 3.41 ± 3.36 |  | 3.05 ± 3.27 |  |
| Life Course Antibiotic Exposure |  |  |  |  |
| *No/Yes, %No* | 93/10 (90%) |  | 88/9 (88%) |  |
| **AAP Exposure (Birth to 6-Months)** | **Mean ± SD** |  | **Mean ± SD** |  |
| PM_10_ (µg/m^3^) | 32.8 ± 5.04*** |  | 29.5 ± 3.61 |  |
| PM_2.5_ (µg/m^3^) | 13.0 ± 1.89*** |  | 11.7± 1.47 |  |
| NO_2_ (ppb) | 20.5 ± 4.70*** |  | 15.08 ± 4.43 |  |

**Supplemental Table 1.** Data are reported as means and standard deviations (SD) unless otherwise noted. Differences between the current sample with complete data (n=103) and those that were excluded due to missing data or being outliers (n=100) were examined using the Welch’s t-tests for normally distributed variables and Wilcoxon tests, for non-normally distributed variables. For categorical variables, chi-square tests were used to test for differences. Statistical significance between participants included in the study and those that were excluded corresponds to ***p < 0.001, **p < 0.01, and *p < 0.05.

**†**Of the 100 participants that were excluded, 90 had available ambient air pollution exposure data, 97 had information available regarding mode of delivery and antibiotic usage, and 95 had available socioeconomic status information.

**Supplemental Table 3.** Average Number of Zeros and Average Predicted Probability of Excess Zeros Within ZINBR Analyses at Each Taxonomic Level

| **Taxonomic Level** | Average Number  of Zeros |  | Average Predicted Probability of Excess Zeros |
| --- | --- | --- | --- |
| Phylum | 77.5 |  | 2.2e-16 |
| Class | 51.0 |  | 0.20 |
| Order | 58.4 |  | 0.36 |
| Family | 53.3 |  | 0.36 |
| Genus | 60.8 |  | 0.42 |

**Supplemental Table 3.** Average number of zeros and average predicted probability of observing an excess at each ample point within ZINBR analyses at each taxonomic level.

**Supplemental Figure 4.** Spearman Correlation Coefficients Between 6-month Postnatal Ambient Air Pollution Exposures and Covariates

**Supplemental Figure 4.** Heatmap of Pearson correlation coefficients between log transformed 6-month postnatal NO_2_, PM_10,_ PM_2.5_ and covariates are reported. A p-value < 0.001, < 0.01 and < 0.05 are denoted by “***”, “**” and “*”, respectively. The socioeconomic status variable is an estimate of socioeconomic status derived from a modified version of the Hollingshead Index.

**Supplemental Table 5.**  Covariation Between 6-month Postnatal Ambient Air Pollution Exposures and Adjusted Covariates

|  | **NO_2_ Exposure** | **PM_2.5_ Exposure** | **PM_10_ Exposure** | **Breastfeeding Per Day** | **Age**  **(days)** | **Birthweight (kg)** | **Socioeconomic Status** |
| --- | --- | --- | --- | --- | --- | --- | --- |
| **NO_2_ Exposure** | 22.08 | 6.73 | 12.41 | -3.42 | -2.62 | 0.45 | 2.90 |
| **PM_2.5_ Exposure** | 6.73 | 3.56 | 7.42 | -0.94 | -2.72 | 0.12 | -0.24 |
| **PM_10_ Exposure** | 12.41 | 7.42 | 25.40 | -2.01 | -9.81 | 0.40 | -3.80 |
| **Breastfeeding Per Day** | -3.42 | -0.94 | -2.01 | 17.07 | -3.08 | -0.29 | -5.84 |
| **Age (days)** | -2.62 | -2.72 | -9.81 | -3.08 | 72.14 | -0.24 | 0.33 |
| **Birthweight (kg)** | 0.45 | 0.12 | 0.40 | -0.29 | -0.24 | 0.16 | -0.11 |
| **Socioeconomic Status** | 2.90 | -0.24 | -3.80 | -5.84 | 0.33 | -0.11 | 132.56 |

**Supplemental Table 5.** Covariation between 6-month postnatal NO_2_, PM_10,_ PM_2.5_exposures and continuous covariates which were included within our models are presented. Continuous covariates include breast feedings per day, infant age in days, infant birthweight (kg) and Hollingshead Index Scores which were used to represent socioeconomic status.
